# Plant Functional Traits Better Explain the Global Latitudinal Patterns of Leaf Insect Herbivory than Climatic Factors

**DOI:** 10.3390/plants14091303

**Published:** 2025-04-25

**Authors:** Yuhui Ji, Xiaoxu Yan, Jiali Xu, Mira Jumak, Ruizhi Zhang, Lan Wang, Jie Gao

**Affiliations:** 1 College of Grassland Science, Xinjiang Agricultural University, Urumqi 830052, China; jiyuhui53@163.com (Y.J.); y1273848445@163.com (X.Y.); 2 College of Life Sciences, Xinjiang Normal University, Urumqi 830054, China; m18099534368@163.com (J.X.); 18999541576@163.com (M.J.); zrz13699361635@163.com (R.Z.); 3 Post-Doctoral Mobile Station, Xinjiang Agricultural University, Urumqi 830052, China

**Keywords:** herbivory, global latitudinal patterns, plant functional traits, climate, soil nutrients

## Abstract

Herbivory reflects the interaction between plants and insects in ecosystems, and its latitudinal patterns at the global scale have attracted widespread attention. While many studies support the latitudinal herbivory hypothesis, it remains contentious. This study, based on a global dataset of 1206 herbivory records, explored the global latitudinal patterns of insect herbivory on leaves and their influencing factors. We found that herbivory decreased with increasing latitude from the equator to the poles, supporting the latitudinal herbivory hypothesis. Latitude affected the variation in climate, soil nutrients, and plant functional traits, which ultimately affected herbivory. Plant functional traits were the key factors affecting the global latitudinal patterns of herbivory, with climatic factors playing an important regulatory role, while soil nutrients had a relatively minor impact, explaining 7.3%, 4.66%, and 0.98% of the latitudinal variation in herbivory, respectively. Specifically, plant height and mean annual temperature were the most important drivers of the global latitudinal patterns of herbivory, explaining 3.39% and 3.03%, respectively. Our study focused on two new perspectives—plant functional traits and soil nutrients. Although soil nutrients had a relatively minor influence on the latitudinal patterns of herbivory, we emphasized the significant impact of plant functional traits on the latitudinal patterns of herbivory. Our findings provide new insights into understanding and predicting the geographic patterns of herbivory and ecological interactions in the context of global climate change, offering important references and ecological significance.

## 1. Introduction

Insect herbivory, the consumption of plants by insects, is one of the most common types of interspecific interactions in nature. The interactions between plants and herbivorous insects affect plant growth, reproduction, and survival, with profound implications for plant community dynamics, ecosystem functions, and biodiversity [1,2,3,4]. Understanding the latitudinal patterns of these interactions, particularly herbivory, is crucial for understanding the mechanisms that control ecosystem functions and predicting their responses to climate change [5]. Herbivory in nature varies greatly across different latitudes, and explaining these latitudinal differences has become one of the central scientific questions in ecology over the past few decades [6,7,8].

The latitudinal herbivory hypothesis (LHH) suggests that tropical regions, with warmer climates, longer growing seasons, and higher species diversity and relative abundance of herbivorous insects, are expected to experience higher levels of herbivory compared to high-latitude regions. According to this theory, the intensity of herbivory (i.e., plant consumption by herbivores) should decrease as latitude increases. However, different studies have failed to reach consistent conclusions on this question [3,9,10,11,12,13]. The latitudinal variation in herbivory is generally considered to result from changes in water, energy, and available resources, which are more favorable in lower latitudes and become harsher in higher latitudes [14]. Increasing evidence suggests that latitudinal patterns of herbivory are primarily driven by underlying gradients of both biotic and abiotic factors [13,15]. However, there is a lack of consistency among studies in explaining the latitudinal variation in herbivory. For example, the latitudinal patterns of herbivory may be entirely dependent on, or independent of, abiotic factors such as temperature and precipitation [16]. To investigate the causes of latitudinal variation in herbivory, it is necessary to analyze this issue from multiple perspectives, considering the roles of both abiotic factors (e.g., climate and soil nutrients) and biotic factors (e.g., plant functional traits).

Climate (especially temperature and precipitation) can influence the species diversity [17], relative abundance [18], and behavioral activity [19] of herbivorous insects, as well as the physiological, phenological, and defensive traits of plants [20,21]. These factors are important drivers of the latitudinal patterns of plant–herbivore interactions [3,22]. Numerous studies have shown that temperature and precipitation generally increase from high to low latitudes, creating more favorable climate conditions, enhancing biodiversity [23], and intensifying biotic interactions [8,10,24,25,26], with plants being attacked by more species and greater numbers of herbivorous insects, leading to increased herbivory [27]. Many studies have confirmed the significant impact of temperature and precipitation on plant–herbivore interactions [14,28,29]. However, Zhang et al. [11] found no significant correlation between precipitation and herbivory. Moreover, previous research has primarily focused on regional scales to examine the effects of climatic factors on plant–herbivore interactions, with varying results. Few studies have addressed the impact of climatic factors on the global latitudinal patterns of herbivory [15,22,30,31].

Soil nutrients influence plant growth, defense, and plant–herbivore interactions, and may be an important ecological factor affecting the latitudinal patterns of herbivory [32]. The nutritional status of soil can affect plant palatability to insects [33]. For example, increased levels of nitrogen (N) and phosphorus (P) can make plant leaves more nutritious [34]. Züst and Agrawal [35] found that herbivorous insects may prefer faster-growing, more nutritious plant individuals. A decline in soil nitrogen availability leads to a reduction in leaf nitrogen concentration, lowering the dietary quality for herbivores (the availability of nitrogen to insects), which in turn reduces insect populations [36]. Soil phosphorus levels can affect leaf nutrient quality and defense levels; increased soil phosphorus improves leaf nutrient quality and reduces plant defenses, making plants more vulnerable to herbivore attacks [37]. Soil pH affects ion concentrations in the soil and the adsorption and mobility of plant nutrients, making it a key factor influencing soil nutrient availability [38]. Higher nutrient availability may also increase herbivore feeding intensity by either increasing plant nutrient content or altering underground interactions [31]. Many studies have demonstrated that plant palatability or leaf nutrient traits are important factors influencing insect herbivory [39], but it remains unclear to what extent soil nutrients influence the global latitudinal variation in herbivory.

In addition, plant functional traits play an important role in regulating herbivory [4]. Li et al. [40] found that plant traits better explain the variation in leaf herbivory rates than environmental factors. The plant apparency hypothesis suggests that plants that are more conspicuous in the community (e.g., tall trees) are more likely to be consumed by herbivores [41]. Zhang et al. [4] also found that higher plant height and larger leaf area were associated with higher herbivory. Herbivory is also related to the leaf morphology of plants [42,43]. For example, plants with hairy leaves tend to experience less herbivory and damage compared to plants with smooth leaves [44]. Additionally, plant–herbivore interactions are influenced by phenological factors. Phenology can affect plant tolerance to herbivory [45], with individuals that flower later in the season typically experiencing lower levels of herbivore damage [46]. Previous studies on the effects of plant functional traits on herbivory have primarily focused on small-scale regional studies, with inconsistent results [15,47,48,49]. However, few studies have examined the influence of plant functional traits on latitudinal variation in herbivory on a global scale [50,51]. Our understanding of how plant functional traits contribute to explaining the global latitudinal patterns of herbivory is still limited.

To explore the global latitudinal patterns of herbivory and their potential driving factors, we built upon the study by Tang et al. [3] by incorporating two new perspectives, namely soil nutrients and plant functional traits, and attempted to analyze the relative contributions of climatic factors, soil nutrients, and plant functional traits to the global latitudinal patterns of herbivory. We hypothesized the following: (1) herbivory decreases with increasing latitude, supporting the latitudinal herbivory hypothesis; (2) plant functional traits and climatic factors are the primary drivers shaping the global latitudinal patterns of herbivory; and (3) soil nutrient factors also play a significant role in driving the global latitudinal patterns of herbivory.

## 2. Materials and Methods

### 2.1. Leaf Insect Herbivory Data

**Figure 1:**
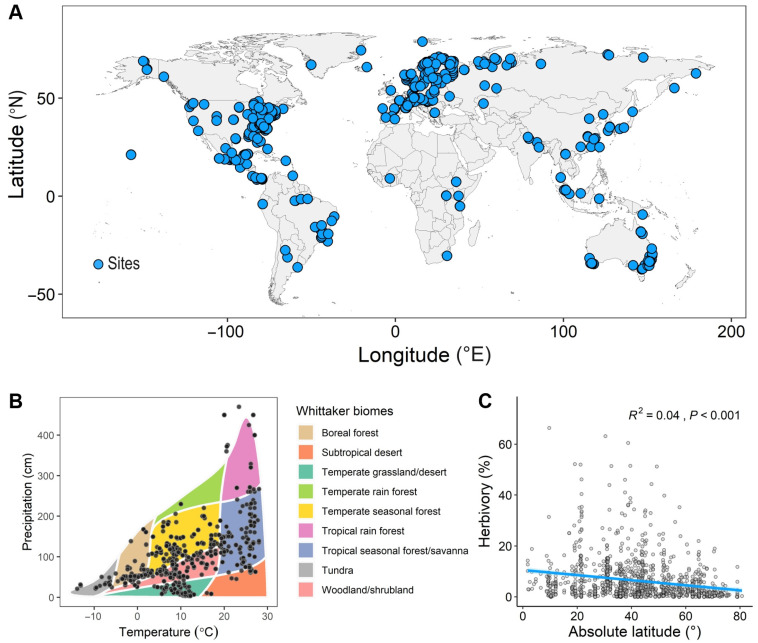
Global distribution of the study sites for herbivory (**A**,**B**) and the global latitudinal patterns in herbivory (**C**). The light blue shaded area represents the 95% confidence interval of the fitted line. *R*^2^ indicates the model’s goodness of fit, and the *p*-value indicates the level of significance.

Building upon the dataset from Tang et al. [3], we incorporated soil nutrient and plant functional trait variables to assemble a dataset of 1206 herbivory data points from 576 species of seed plants across 110 plant families (Figure 1A,B). This dataset included 691 herbivory records for woody plants and 515 for non-woody plants. These herbivory data were quantitatively synthesized from earlier studies, including 685 herbivory data points from 292 locations collected by Zhang et al. [11], 33 data points from 19 locations collected by Mendes et al. [52], and 488 data points from 199 locations collected by Tang et al. [3]. The main criteria for collecting leaf herbivory data were as follows: (1) herbivory data were collected from leaves of each plant species, rather than flowers, seeds, or underground plant parts; (2) only data from field observations of leaf herbivory were included, excluding those from greenhouse or laboratory experiments; (3) only herbivory data that represented the majority of leaf damage throughout the leaf’s life cycle were included, such as cumulative damage to mature leaves and leaf herbivory data collected at the end of the growing season; and (4) only data with clearly defined research site coordinates and species information were included. Leaf herbivory was measured as the percentage of leaf area consumed by insects. Insect herbivory includes chewing, sucking, mining, gall-forming, and scraping.

### 2.2. Climate and Soil Data

We extracted the mean annual temperature (MAT) and mean annual precipitation (MAP) data at a 1 km spatial resolution from the WorldClim global climate database (https://www.worldclim.org/, accessed on 20 November 2023) based on the latitude and longitude information in the dataset. Additionally, we retrieved soil data, including soil total nitrogen (N) content, total phosphorus (P) content, and soil pH at the top 30 cm layer, from the SoilGrids database (https://soilgrids.org/, accessed on 1 December 2023), with a resolution of 250 m.

### 2.3. Plant Functional Traits Data

We obtained the average trait data for each plant species by searching authoritative plant databases, which primarily included morphological traits such as plant height, leaf size, leaf shape, leaf margin, and leaf texture, as well as phenological data on flowering and fruiting. The plant databases we used included World Flora Online (https://www.worldfloraonline.org), Plants of the World Online (http://powo.science.kew.org/), and Euro + Med PlantBase (https://europlusmed.org/), accessed between 1 October and 30 November 2023. Due to the difficulty of obtaining the actual trait values for species at specific locations, we used the average trait values for each species as the plant functional trait variables in this study. Leaf size, defined as leaf area, was calculated using the formula (Leaf Area = Leaf Length × Leaf Width × 0.75) based on the leaf length and width (both in cm) obtained from the searches [53,54,55]. Since there is significant morphological variation among different leaves of the same plant, it is difficult to precisely categorize leaf margin and leaf texture, so we simplified the classification: leaf margin was divided into entire and non-entire (including serrated, toothed, shallowly lobed, and deeply lobed, etc.), and leaf texture was classified as leathery or non-leathery (e.g., papery, membranous, and herbaceous). Leaf shape was classified according to the primary shape of the leaves, including ovate, elliptical, lanceolate, or linear, etc. Additionally, we verified the leaf morphology of each species through the Plants of the World Online database (https://powo.science.kew.org/, accessed between 1 October and 30 November 2023) to ensure the accuracy of the leaf morphology data. Flowering and fruiting phenology were calculated based on the months described in the plant descriptions, with a simple conversion to days. For example, if flowering occurs from March to May, the flowering phenology period is represented by the number of days between 1 March and 31 May, and the same method was applied to calculate the fruiting phenology period. In this study, the flowering and fruiting phenology refers to the duration of the flowering and fruiting stages.

### 2.4. Statistical Analysis

All data analyses in this study were conducted in R v4.3.1 (https://www.r-project.org/, accessed on 10 December 2023). A linear regression model was used to fit the relationship between herbivory and latitude to explore the latitudinal patterns of herbivory from the tropics to the poles, and to assess whether it conforms to the LHH. Latitude in the southern hemisphere is typically represented by negative values, so in this study, we used absolute latitude to represent latitude. We then performed similar linear regression analyses to test the latitudinal patterns of climate variables (MAT, MAP), soil nutrient variables (Soil N, Soil P, and Soil pH), and plant functional traits (plant height, leaf area, flowering phenology, and fruiting phenology), and fitted linear regression models to examine the relationships between these variables and herbivory in order to analyze their effects on the latitudinal patterns of herbivory. The detailed fitting parameters of each linear regression model can be found in Table S1. The *R*^2^ value represents the goodness-of-fit of the models, and the *p*-value indicates statistical significance.

We performed variation partitioning and the hierarchical partitioning of explanatory variables for the latitudinal spatial variation in herbivory using the “rdacca.hp” package [56] to quantify the relative importance of groups of explanatory variables (climate, soil, and functional traits) and individual explanatory variables (MAT, MAP, soil N, soil P, soil pH, plant height, leaf area, leaf shapes, leaf margin, leaf texture, flowering phenology, and fruiting phenology). To address potential multicollinearity among these explanatory variables, we conducted multivariate correlation analysis using the “linkET” package in R to assess the interrelationships among the explanatory variables.

A piecewise structural equation model (piecewise SEM) was used to explore the pathways through which climatic factors, soil nutrients, and plant functional traits influence the latitudinal spatial variation in herbivory. To evaluate the robustness of the relationships between key ecological factors and herbivory, we used piecewise SEM to account for the random effects of sampling sites and provide the “marginal” and “conditional” contributions of environmental predictor variables. These analyses were conducted in R using the “piecewiseSEM”, “nlme”, and “lme4” packages. The goodness-of-fit of the models was assessed using Fisher’s *C* test. Based on the acceptable model criteria, i.e., a significance level of *p* < 0.05 and optimal model fit (0 ≤ Fisher’s *C*/*df* ≤ 2 and 0.05 < *p* ≤ 1.00), the models were stepwise refined and improved to select the best-fitting model.

## 3. Results

### 3.1. Global Latitudinal Patterns of Herbivory

The average herbivory level for all species was 6.33% (SE = 0.24, n = 1206). Our results showed that herbivory decreased with increasing latitude (*R*^2^ = 0.04, *p* < 0.001, Figure 1C), meaning that herbivory gradually declined from the equator to the poles, supporting the LHH.

### 3.2. Impact of Climate on the Global Latitudinal Patterns of Herbivory

**Figure 2:**
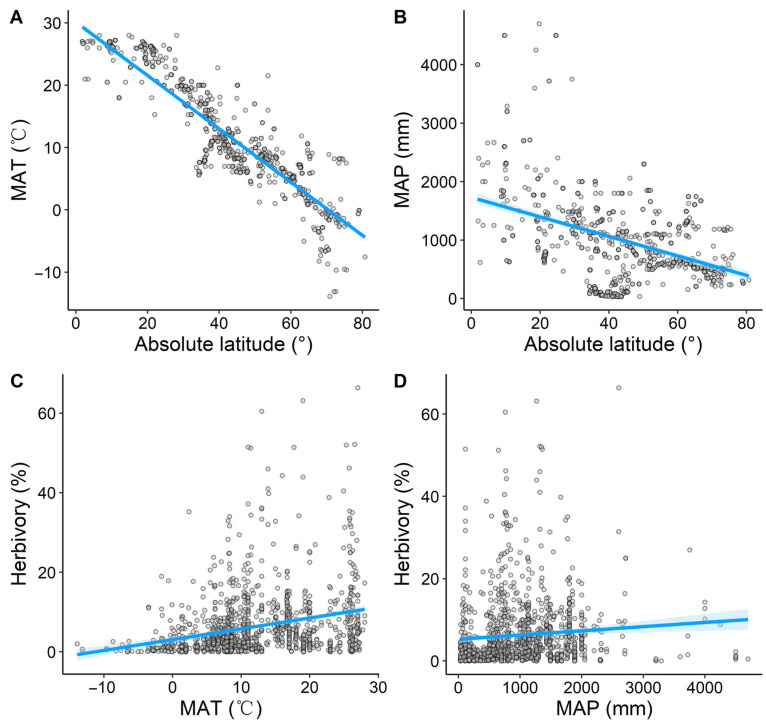
The latitudinal distribution of climate (**A**,**B**) and the effects of climatic factors on herbivory (**C**,**D**). Climatic factors include mean annual temperature (MAT) and mean annual precipitation (MAP). The light blue shaded area represents the 95% confidence interval of the fitted line.

Both MAT and MAP decreased significantly with increasing latitude (*R*^2^ = 0.81, *p* < 0.001, Figure 2A; *R*^2^ = 0.17, *p* < 0.001, Figure 2B), indicating that temperature and precipitation decreased from the equator toward the poles. Herbivory increased significantly with MAT (*R*^2^ = 0.07, *p* < 0.001, Figure 2C), and there was a weak positive correlation between herbivory and MAP (*R*^2^ = 0.007, *p* = 0.003, Figure 2D). Compared to the latitudinal pattern of MAP, the latitudinal pattern of MAT was more pronounced, which may explain why the positive correlation between herbivory and MAT was stronger than that between herbivory and MAP.

### 3.3. Impact of Soil Nutrients on the Global Latitudinal Patterns of Herbivory

**Figure 3:**
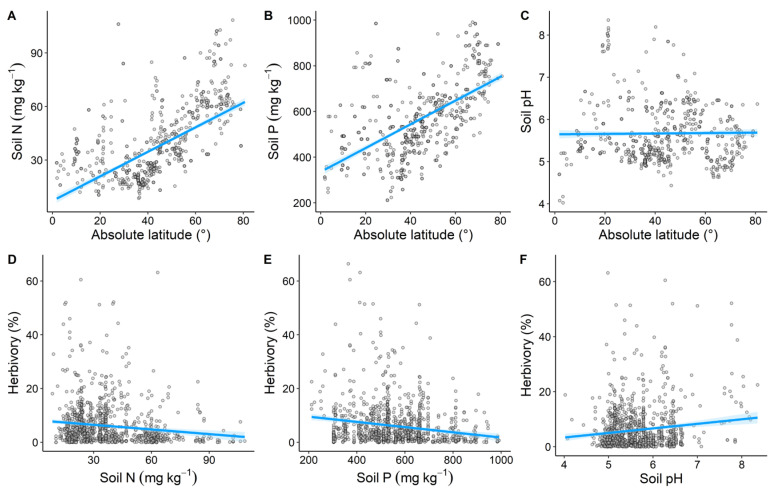
The latitudinal distribution of soil nutrients (**A**–**C**) and the effects of soil nutrient factors on herbivory (**D**–**F**). Soil nutrient factors include soil total nitrogen content (Soil N), soil total phosphorus content (Soil P), and soil pH. The light blue shaded area represents the 95% confidence interval of the fitted line.

Both soil N and P increased significantly with increasing latitude (*R*^2^ = 0.38, *p* < 0.001, Figure 3A; *R*^2^ = 0.35, *p* < 0.001, Figure 3B), while soil pH showed no significant trend (*R*^2^ < 0.01, *p* = 0.663, Figure 3C). Herbivory decreased significantly with increasing soil N and P (*R*^2^ = 0.02, *p* < 0.001, Figure 3D; *R*^2^ = 0.03, *p* < 0.001, Figure 3E), but increased significantly with increasing soil pH (*R*^2^ = 0.02, *p* < 0.001, Figure 3F).

### 3.4. Impact of Plant Functional Traits on the Global Latitudinal Patterns of Herbivory

**Figure 4:**
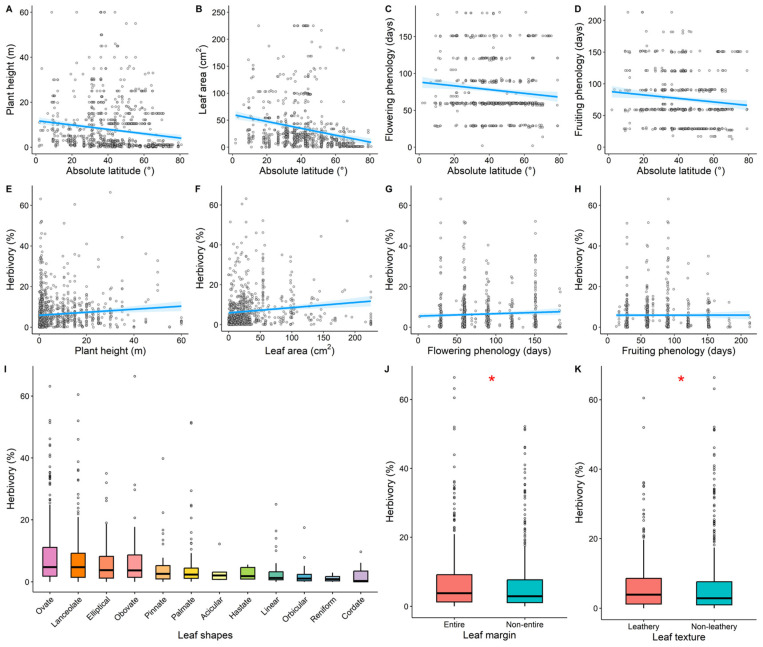
The latitudinal distribution of plant functional traits (**A**–**D**) and the effects of plant functional traits on herbivory (**E**–**K**). The light blue shaded area represents the 95% confidence interval of the fitted line. Asterisk indicates levels of significance (* *p* < 0.05).

Plant height (*R*^2^ = 0.02, *p* < 0.001, Figure 4A), leaf area (*R*^2^ = 0.06, *p* < 0.001, Figure 4B), flowering phenology (*R*^2^ = 0.01, *p* = 0.004, Figure 4C), and fruiting phenology (*R*^2^ = 0.01, *p* = 0.008, Figure 4D) all showed significant decreasing trends with increasing latitude. Herbivory increased significantly with plant height and leaf area (*R*^2^ = 0.009, *p* = 0.001, Figure 4E; *R*^2^ = 0.02, *p* < 0.001, Figure 4F). No significant changes were observed between herbivory and flowering or fruiting phenology (*R*^2^ < 0.01, *p* > 0.05, Figure 4G,H). Additionally, herbivory varied across leaf shapes, with the highest herbivory observed in ovate leaves, followed by lanceolate, elliptical, and obovate leaves, while herbivory was relatively lower in reniform and cordate leaves. Herbivory was significantly higher in entire-margined leaves compared to non-entire-margined leaves (*p* < 0.05, Figure 4J), and significantly higher in leathery leaves compared to non-leathery leaves (*p* < 0.05, Figure 4K).

### 3.5. Relative Importance of Climate, Soil Nutrients, and Plant Functional Traits in Predicting Latitudinal Variation in Herbivory

**Figure 5:**
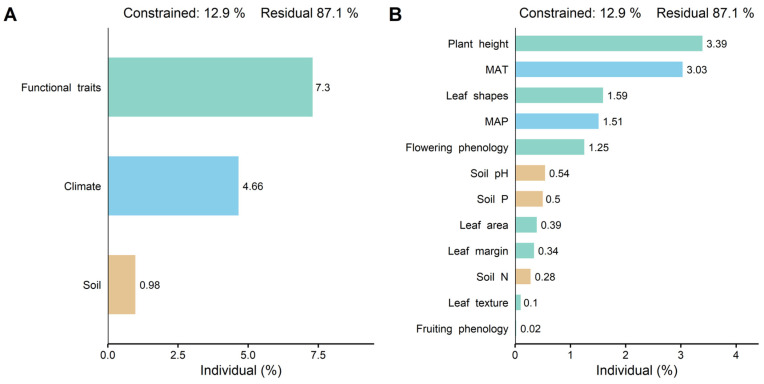
The relative importance of predictor groups (**A**) and individual predictors (**B**) on herbivory. These factors collectively explain 12.9% of the global latitudinal variation in herbivory. The individual effect of each variable factor (from hierarchical partitioning) is equal to the marginal effect of that variable factor plus the average allocated value of its shared common effect with other variable factors. MAT, mean annual temperature; MAP, mean annual precipitation; Soil N, soil total nitrogen content; and Soil P, soil total phosphorus content.

**Figure 6:**
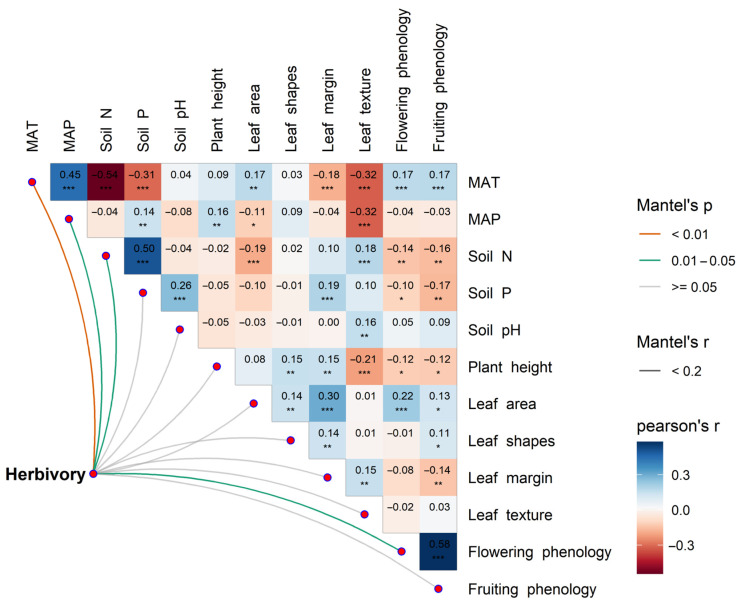
Multivariate correlation analysis among potential influencing factors for herbivory. MAT, mean annual temperature; MAP, mean annual precipitation; Soil N, soil total nitrogen content; and Soil P, soil total phosphorus content. Asterisks indicate levels of significance (*** *p* < 0.001; ** *p* < 0.01; and * *p* < 0.05).

**Figure 7:**
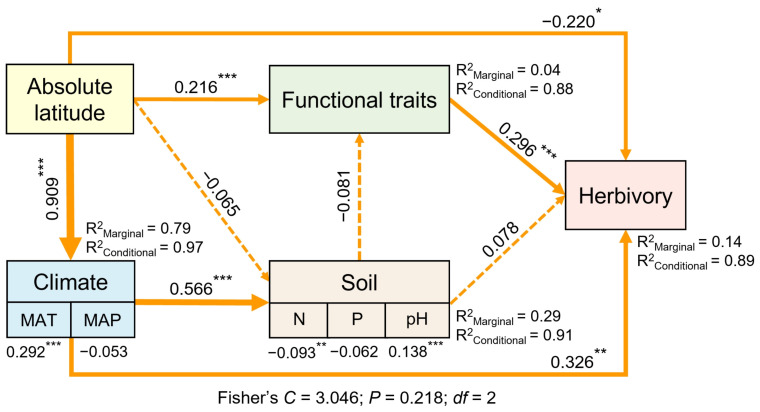
Structural equation modeling illustrates how latitude affects variation in herbivory. Arrows represent hypothesized impact pathways, with numbers next to the arrows indicating standardized path coefficients, and asterisks denoting levels of significance (*** *p* < 0.001; ** *p* < 0.01; and * *p* < 0.05). The thickness of the arrows represents the relative magnitude of the path coefficients. *R*^2^ represents the proportion of variance for each explanatory variable.

Variation partitioning and hierarchical partitioning analyses revealed that plant functional traits accounted for the most variation in the latitudinal patterns of herbivory, followed by climatic factors, while soil factors contributed the least (Figure 5A). Further analysis of the independent contributions of individual explanatory variables to the latitudinal variation in herbivory indicated that plant height had the greatest independent contribution, followed by MAT (Figure 5B). Among the climatic factors, MAT explained more of the latitudinal variation in herbivory than MAP. Overall, climate factors, soil nutrient factors, and functional traits have a significant impact on insect herbivory (Figure 6). The piecewise SEM further indicated that latitude not only directly affects herbivory, but also influences it indirectly by affecting climate factors and plant functional traits (Figure 7).

## 4. Discussion

### 4.1. Global Latitudinal Patterns of Herbivory and Their Influencing Factors

Our study found that insect herbivory on leaves significantly decreased with increasing latitude, showing a decline from low latitudes to high latitudes (Figure 1C), which supports the LHH. This result is consistent with the findings of Kozlov et al. [10] and Zhang et al. [11]. The distribution pattern of herbivory decreasing with increasing latitude is generally considered to be the result of the combined effect of multiple factors [3,10,57,58]. Previous studies have focused more on the impact of climatic factors and plant chemical defenses on herbivory, with many studies suggesting that climate is the primary driver of global herbivory variation [3,58]. However, our study found that plant functional traits were more effective than climatic factors in explaining the global latitudinal variation in herbivory, while the effect of soil nutrient factors on herbivory variation was weaker (Figure 5 and Figure 7). The variation in plant functional traits and climate along the latitudinal gradient drove the global latitudinal pattern of herbivory (Figure 2, Figure 4 and Figure 7). These findings provide a new perspective on understanding biological interactions under global climate change and are of great significance for understanding and predicting the geographic patterns of herbivory in the context of future climate change.

### 4.2. Effects of Climatic Factors on Latitudinal Variation in Herbivory

Our study found that latitudinal variation in herbivory was significantly affected by climatic factors, showing an increase in both MAT and MAP (Figure 2). This is consistent with the findings of Tang et al. [3]. As cold-blooded animals (poikilotherms), herbivorous insects are highly dependent on external environmental conditions, especially temperature and precipitation [59]. Temperature and precipitation can directly affect the metabolism, growth, development, reproduction, and behavior of herbivorous insects [28,60], and also influence plant growing seasons and plant traits [61]. In tropical regions with ample water and heat, plants generally exhibit higher species richness and longer growing seasons, featuring evergreen broadleaf species that provide both habitats for insect settlement and oviposition, and abundant food resources for insect growth and survival [62,63]. Additionally, herbivorous insects in tropical regions have more active metabolic activity. To meet the energy requirements for growth and metabolism, insects increase their feeding intensity on plant leaves, leading to higher herbivory in the low-latitude tropics [64]. However, as latitude increases, both MAT and MAP decrease (Figure 2), water and heat conditions change, and the environment for survival becomes relatively harsh, reducing the frequency and intensity of biological interactions [65,66], which in turn decreases herbivory.

Compared to MAP, MAT showed a stronger correlation with herbivory and a greater independent explanatory ability for latitudinal variation in herbivory (Figure 2 and Figure 5). Zhang et al. [11] found that temperature is a better predictor of herbivory variation than precipitation, as it is significantly positively correlated with herbivory, which is consistent with our findings. While sufficient precipitation supports plant growth and provides more food resources for insects [67,68], excessive rainfall may increase leaf moisture, making it less favorable for insect feeding [3]. Additionally, compared to MAP, the negative correlation between MAT and latitude was stronger (Figure 2), which may also explain why MAT played a more important role in shaping the latitudinal patterns of herbivory.

### 4.3. Effects of Soil Nutrient Factors on Latitudinal Variation in Herbivory

Soil nutrient factors had a weaker effect on latitudinal variation in herbivory compared to climatic factors (Figure 5), which is somewhat contrary to our hypothesis. This may be because climate conditions have a more direct and significant impact on the physiology and behavior of insects [69,70], while the direct effects of soil nutrients on insects are relatively weaker. Soil nutrients can directly affect plant nutrition and defense levels, which in turn indirectly influence plant–insect interactions [71].

A recent global experimental study showed that adding nitrogen and phosphorus increases herbivory by invertebrates [72], whereas our findings present a contrasting result. We found that soil nitrogen and phosphorus contents increased significantly with latitude, and herbivory decreased significantly with increasing soil nitrogen and phosphorus contents (Figure 3), which was consistent with the general pattern of herbivory decreasing with latitude. This suggests that spatial variation in the soil nutrient content across latitudes may contribute to the latitudinal variation in herbivory. Increased nitrogen and phosphorus contents in the soil improve plant nutritional status, thereby affecting herbivorous insect host selection, growth, development, and reproductive capacity [13]. Under nutrient-rich soil conditions, plants may allocate more resources to defensive traits, such as the production of secondary metabolites, which may negatively impact insect herbivory [73,74]. Additionally, we found that herbivory increased significantly with increasing soil pH. In acidic soils, plants tend to produce more secondary metabolites such as phenolics and flavonoids, which inhibit insect herbivory [58]. In the context of global climate change, changes in the soil nutrient status may further intensify insect herbivory, which could have profound long-term impacts on ecosystem functions [58].

### 4.4. Effects of Plant Functional Traits on Latitudinal Variation in Herbivory

Our results showed that plant functional traits explained the latitudinal variation in herbivory better than climatic and soil nutrient factors (Figure 5A), which differed from the findings of Tang et al. [3] and Liu et al. [58]. Plant functional traits directly determine the intensity and nature of plant–insect interactions, which can better explain the spatial variation in herbivory [39]. We found that plant height had the strongest independent explanatory power for latitudinal variation in herbivory, followed by leaf shape and flowering phenology (Figure 5B). Taller plants typically have more biomass and nutrient reserves, providing abundant food resources and good habitats for insects. Their relatively lower defense levels make them more susceptible to herbivory [39,50,75,76]. Our study found that herbivory increased significantly with plant height (Figure 4), which was consistent with the findings of Zhang et al. [51] and Wang et al. [49].

Leaf shape, leaf margin, and leaf texture are key traits affecting plant physical defenses [39,44,77]. Some insects are adapted to feeding and reproducing on plants with specific leaf shapes, margins, or textures, which strengthens the impact of plant traits on herbivory [39,78]. In our study, we found that leathery leaves experienced significantly higher levels of herbivory than non-leathery leaves. This pattern may be related to their mechanical properties and chemical composition. Leathery leaves typically possess a thicker cuticle and higher concentrations of secondary metabolites (such as tannins and alkaloids), which may attract certain specialist herbivores—particularly insects that have adapted to tolerate or even utilize these compounds, such as some caterpillars and beetles [79,80]. In contrast, other herbivores may prefer non-leathery leaves due to their softer texture and easier digestibility. We also observed that entire leaves exhibited higher herbivory than non-entire leaves. This may be linked to the leaf margin structure and insect feeding behaviors. Entire margins can offer a more accessible surface area, while lobed or irregular margins may reduce feeding efficiency [81]. Additionally, intact margins may be more appealing to herbivores, despite potentially lower defensive efficacy [82]. Overall, different leaf types may influence herbivore preferences through a combination of plant defense traits and herbivore adaptations [83,84]. Our findings likely reflect the interaction of these complex ecological and biological factors.

Additionally, during flowering, plants often allocate limited resources to flower production and maintenance, reducing the leaf nutrient content and the synthesis of chemical defenses [85]. The reduction in the leaf nutrient content may decrease herbivory intensity [39], but the weakening of defenses could make the leaves more susceptible to insect attacks [86]. These contrasting influences may weaken the trend of herbivory variation with flowering duration (Figure 4G). Differences in leaf longevity can indeed affect the assessment of damage rates. If the variation in leaf longevity across species is not considered, it may lead to misjudgments regarding the damage rates of species with short-lived leaves. In our study, we did not directly incorporate leaf longevity as a factor, which may introduce some bias. This is an issue we plan to address in future research.

## 5. Conclusions

Our study analyzed the global latitudinal patterns of herbivory and their influencing factors. We found that herbivory decreased with increasing latitude, supporting the LHH. Latitude primarily influenced herbivory through its effects on climate and plant functional traits. Latitudinal variation in herbivory was mainly driven by plant functional traits (7.3%) and climate (4.66%), with plant height and MAT being the strongest explanatory factors, accounting for 3.39% and 3.03%, respectively. Soil nutrient factors had a relatively minor impact (0.98%). Our study highlights the importance of plant functional traits in explaining the global latitudinal patterns of herbivory, which is crucial for understanding the ecological processes in species interactions. These findings emphasize the need to consider plant functional traits in future studies on spatial variation in herbivory, offering new insights into the mechanisms and patterns of multi-trophic interactions in the context of global climate change.

## Data Availability

That can be provided upon request. The data are not publicly available at this time because a portion of them is being used in another manuscript we are currently preparing for submission.

## Supplementary Materials

The following supporting information can be downloaded at: https://www.mdpi.com/article/10.3390/plants14091303/s1, Table S1: Parameters of linear regression model.
