# Supplementary material for: Plant Functional Traits Better Explain the Global Latitudinal Patterns of Leaf Insect Herbivory than Climatic Factors
Source: Plants (Basel). 2025 Apr 25;14(9):1303. doi: 10.3390/plants14091303 (PMC12073595; doi:10.3390/plants14091303)
Supplement: Supplementary file 1 [file plants-14-01303-s001.zip › plants-3552734-supplementary.pdf]

# Supplementary Materials

**Table S1.** Parameters of linear regression model. MAT, mean annual temperature; MAP, mean annual precipitation; Soil N, soil total nitrogen content; Soil P, soil total phosphorus content. Level of significance: * *P* < 0.05; ** *P* < 0.0l; *** *P* < 0.00l.

| Parameters | Independent Variable | Estimate | Std. Error | t value | Pr (>\|t\|) | df | F | $R^2$ | *p*-value | AIC |
|---|---|---|---|---|---|---|---|---|---|---|
| Herbivory | Absolute latitude | -0.100 | 0.014 | -7.348 | 3.7e-13 *** | 1204 | 53.990 | 0.043 | 3.705e-13 | 8495.465 |
| | (Intercept) | 10.579 | 0.625 | 16.930 | < 2e-16 *** | | | | | |
| Herbivory | MAT | 0.272 | 0.028 | 9.617 | < 2e-16 *** | 1204 | 92.490 | 0.071 | < 2.2e-16 | 8459.108 |
| | (Intercept) | 3.070 | 0.411 | 7.478 | 1.45e-13 *** | | | | | |
| Herbivory | MAP | 0.001 | 0.000 | 2.964 | 0.003 ** | 1204 | 8.784 | 0.007 | 0.003 | 8539.602 |
| | (Intercept) | 5.281 | 0.426 | 12.385 | < 2e-16 *** | | | | | |
| Herbivory | Soil N | -0.058 | 0.013 | -4.473 | 8.55e-06 *** | 1066 | 20.000 | 0.018 | 8.554e-06 | 7464.343 |
| | (Intercept) | 8.246 | 0.533 | 15.465 | < 2e-16 *** | | | | | |
| Herbivory | Soil P | -0.010 | 0.002 | -6.021 | 2.36e-09 *** | 1100 | 36.250 | 0.032 | 2.357e-09 | 7699.071 |
| | (Intercept) | 11.479 | 0.934 | 12.287 | < 2e-16 *** | | | | | |
| Herbivory | Soil pH | 1.655 | 0.394 | 4.205 | 2.83e-05 *** | 1066 | 17.680 | 0.016 | 2.828e-05 | 7466.628 |
| | (Intercept) | -3.259 | 2.245 | -1.452 | 0.147 | | | | | |
| Herbivory | Plant height | 0.075 | 0.023 | 3.226 | 0.0012 ** | 1103 | 10.410 | 0.009 | 0.001 | 7826.620 |
| | (Intercept) | 5.885 | 0.308 | 19.100 | < 2e-16 *** | | | | | |
| Herbivory | Leaf area | 0.026 | 0.007 | 3.850 | 0.0001 *** | 865 | 14.820 | 0.017 | 0.0001 | 6244.631 |
| | (Intercept) | 5.910 | 0.376 | 15.720 | < 2e-16 *** | | | | | |
| Herbivory | Flowering phenology | 0.012 | 0.008 | 1.510 | 0.131 | 771 | 2.281 | 0.003 | 0.131 | 5511.764 |
| | (Intercept) | 5.498 | 0.682 | 8.059 | 2.93e-15 *** | | | | | |
| Herbivory | Fruiting phenology | 1.587e-05 | 7.626e-03 | 0.002 | 0.998 | 638 | 4.328e-06 | 6.783e-09 | 0.998 | 4519.126 |
| | (Intercept) | 5.956e+00 | 6.653e-01 | 8.953 | < 2e-16 *** | | | | | |
| MAT | Absolute latitude | -0.428 | 0.006 | -71.850 | < 2e-16 *** | 1204 | 5163.000 | 0.811 | < 2.2e-16 | 6494.379 |
| | (Intercept) | 30.107 | 0.273 | 110.450 | < 2e-16 *** | | | | | |
| MAP | Absolute latitude | -16.677 | 1.058 | -15.760 | < 2e-16 *** | 1204 | 248.300 | 0.171 | < 2.2e-16 | 18991.345 |
| | (Intercept) | 1731.355 | 48.489 | 35.710 | < 2e-16 *** | | | | | |
| Soil N | Absolute latitude | 0.686 | 0.027 | 25.490 | < 2e-16 *** | 1066 | 649.900 | 0.379 | < 2.2e-16 | 8803.443 |
| | (Intercept) | 7.185 | 1.247 | 5.760 | 1.1e-08 *** | | | | | |

| | | | | | | | | | | |
|---|---|---|---|---|---|---|---|---|---|---|
| Soil P | Absolute latitude | 5.195 | 0.212 | 24.480 | < 2e-16 *** | 1100 | 599.100 | 0.353 | < 2.2e-16 | 13676.629 |
| | (Intercept) | 336.025 | 9.860 | 34.080 | < 2e-16 *** | | | | | |
| Soil pH | Absolute latitude | 0.000 | 0.001 | 0.436 | 0.663 | 1066 | 0.190 | 0.000 | 0.663 | 2012.167 |
| | (Intercept) | 5.647 | 0.052 | 108.814 | < 2e-16 *** | | | | | |
| Plant height | Absolute latitude | -0.098 | 0.019 | -5.207 | 2.29e-07 *** | 1103 | 27.110 | 0.024 | 2.288e-07 | 8378.064 |
| | (Intercept) | 11.838 | 0.854 | 13.869 | < 2e-16 *** | | | | | |
| Leaf area | Absolute latitude | -0.636 | 0.087 | -7.311 | 6.04e-13 *** | 865 | 53.450 | 0.058 | 6.041e-13 | 8998.072 |
| | (Intercept) | 60.387 | 3.955 | 15.269 | < 2e-16 *** | | | | | |
| Flowering phenology | Absolute latitude | -0.254 | 0.087 | -2.919 | 0.004 ** | 771 | 8.518 | 0.011 | 0.004 | 7855.975 |
| | (Intercept) | 88.291 | 3.951 | 22.348 | < 2e-16 *** | | | | | |
| Fruiting phenology | Absolute latitude | -0.278 | 0.104 | -2.670 | 0.008 ** | 638 | 7.132 | 0.011 | 0.008 | 6620.138 |
| | (Intercept) | 88.099 | 4.806 | 18.33 | < 2e-16 *** | | | | | |
